# From child maltreatment to ICD‐11 complex post‐traumatic stress symptoms: The role of emotion regulation and re‐victimisation

**DOI:** 10.1002/jclp.22655

**Published:** 2018-06-22

**Authors:** Matthias Knefel, Brigitte Lueger‐Schuster, Thanos Karatzias, Mark Shevlin, Phil Hyland

**Affiliations:** ^1^ Department of Applied Psychology: Health, Development, Enhancement and Intervention Faculty of Psychology, University of Vienna Vienna Austria; ^2^ School of Health & Social Care Edinburgh Napier University Edinburgh UK; ^3^ NHS Lothian, Rivers Centre for Traumatic Stress Edinburgh UK; ^4^ School of Psychology Ulster University Derry UK; ^5^ School of Business National College of Ireland Dublin Ireland

**Keywords:** child maltreatment, complex PTSD, emotion regulation, ICD‐11, path analysis

## Abstract

**Objective:**

Complex post‐traumatic stress disorder (CPTSD) will be introduced in ICD‐11 and comprises symptoms of PTSD and disturbances in self‐organisation (DSO). The association of trauma with PTSD and DSO is not yet fully understood. We investigated the path from child maltreatment to PTSD and DSO and examined the mediating role of emotion regulation (ER) and adult interpersonal re‐victimisation.

**Method:**

Adult patients (*N* = 193) from a Scottish National Health Service clinic participated in the project. Participants completed measures of life events, ICD‐11 PTSD and CPTSD, and ER. Path analysis was used to assess possible direct and indirect effects from childhood trauma on current post‐traumatic psychopathology.

**Results:**

Overall results indicate that the path from child maltreatment to PTSD is a direct one, while the path to DSO is indirectly mediated by ER.

**Conclusions:**

Future research should address the potentially beneficial effect of treatment protocols for CPTSD explicitly aiming at reducing ER difficulties.

## 1 INTRODUCTION

The ICD‐11 workgroup on the classification of disorders specifically associated with stress (Maercker et al., 2013) proposed a new diagnosis, complex post‐traumatic stress disorder (CPTSD). This new diagnosis comprises the core symptoms of PTSD (re‐experiencing, avoidance, and sense of ongoing threat) and additionally includes symptoms of disturbances in self‐organisation (DSO; affect dysregulation, negative self‐concept, and disturbances in relationships). Typically, CPTSD is associated with prolonged interpersonal trauma such as child maltreatment or torture (e.g. Karatzias et al., 2017), however, the ICD‐11 proposal does not specify a certain type of trauma as prerequisite (Maercker et al., 2013). So far, research found some support for the distinction between PTSD and CPTSD (Brewin et al., 2017). However, the path from different types of prolonged interpersonal trauma to PTSD and DSO is not yet fully understood. This study is the first ever to test the role of emotion regulation (ER) as mediating factor from child maltreatment to PTSD and DSO as per proposed ICD‐11 criteria.

The concept of CPTSD has long been subject to clinical and scientific debate. This is rooted in the observation that certain types of traumatic experiences that occur repeatedly and cumulatively, usually over a period of time and within specific relationships and contexts (complex trauma) are associated with other but the typical PTSD symptoms (Herman, 1992). However, based on the field trials (Roth, Newman, Pelcovitz, van der Kolk, & Mandel, 1997), DSM‐IV (American Psychiatric Association, 2000) did not include CPTSD as a separate diagnostic entity, but rather as a ‘associated features’ of PTSD, termed disorders of extreme stress not otherwise specified (DESNOS). In *ICD‐10*, disturbances related to complex trauma are classified under the diagnostic term enduring personality change after catastrophic experience (World Health Organization, 1992). However, this construct failed to attract scientific or clinical interest (Maercker et al., 2013). More recently, the DSM‐5 workgroup on PTSD decided to not include CPTSD as a separate diagnosis (Friedman et al., 2011), based on a critical evaluation of the existing literature pointing out a lack of conceptual clarity and validity (Resick et al., 2012). A considerable scientific effort was made in the last years by advocates of CPTSD aiming to substantiate the concept, assessment measures, and clinical characteristics of CPTSD (Brewin et al., 2017). The ICD‐11 workgroup decided to include CPTSD in their proposal for the classification of disorders specifically associated with stress (Maercker et al., 2013). CPTSD as proposed for ICD‐11 included symptoms of PTSD and DSO and has been shown to capture the symptom presentation of survivors of more complex interpersonal trauma, such as child maltreatment (Cloitre, Garvert, Brewin, Bryant, & Maercker, 2013). Typically, child maltreatment is associated with a broad range of psychopathological sequelae (D'Andrea, Ford, Stolbach, Spinazzola, & Van der Kolk, 2012). These detrimental consequences are likely to be related to neurodevelopmental adaptions to child maltreatment, which are at the neurobiological basis of a range of mental health problems (Teicher & Samson, 2016).

A prominent, trans‐diagnostic problem associated with neurobiological changes due to interpersonal trauma such as child maltreatment is ER (McLaughlin, Peverill, Gold, Alves, & Sheridan, 2015). ER plays an important role in the development and maintenance of various mental disorders (Berking & Wupperman, 2012) including PTSD (Cloitre, Miranda, Stovall‐McClough, & Han, 2005). The ability to regulate one's emotional reactions is shaped during the development from childhood to adulthood (Thompson & Goodman, 2010) and influenced by intrinsic and extrinsic factors (Fox & Calkins, 2003). Child maltreatment, including child abuse and neglect, is associated with a wide range of psychopathological sequelae throughout the lifespan with impaired ER abilities being a core feature that may account for this heightened risk of mental disorders (Dvir, Ford, Hill, & Frazier, 2014).

The development of PTSD following the experience of traumatic events is influenced by a variety of factors (Ozer, Best, Lipsey, & Weiss, 2003). Among these, the ability to manage intense emotional reactions and to accomplish one's goals, termed ER, seems of particular interest for adult survivors of child maltreatment. Caregivers play a crucial role in the development of ER abilities (Thompson, 2011) and children who are maltreated by their caregivers do not only lack support in managing their feelings in everyday life, but also have to deal with negative emotions typically associated with maltreatment. Child maltreatment disrupts the acquisition of functional ER strategies (Dvir et al., 2014), which in turn are associated with symptoms of PTSD that involve intense negative emotions (Kaczkurkin et al., 2017). ER was also found to mediate the relationship between child maltreatment and symptom complexity in previous research (Choi, Choi, Gim, Park, & Park, 2014; Stevens et al., 2013). However, various aspects of ER seem to be differentially associated with the dimensions of post‐traumatic symptomatology (Tull, Barrett, McMillan, & Roemer, 2007) and with characteristics of traumatic experiences. Early onset interpersonal trauma, such as child maltreatment, seems to be stronger associated with dysfunctional aspects of ER than other types of trauma (Ehring & Quack, 2010) and different types of child maltreatment seem to be associated with different types of symptomatology and ER (Petrenko, Friend, Garrido, Taussig, & Culhane, 2012). Emotional abuse was found to be more associated with aspects of ER characterised by inappropriate or impulsive reactions to emotional situations, while emotional neglect was more associated with aspects of ER related to poorer understanding of emotions (Berzenski, 2018). Neglected children seem to have more difficulties discriminating emotions than abused children and tend to react hopeless in stressful situations, while abused children tend to be more angry (Hildyard & Wolfe, 2002). These different aspects of ER predicted different facets of adult maladaptation in terms of psychopathology and interpersonal problems (Berzenski, 2018). Which aspects of ER contribute to CPTSD, that is either PTSD or DSO, have not yet been explored.

Survivors of child maltreatment are also at risk of being re‐victimised in adulthood (Schumm, Hobfoll, & Keogh, 2004). Widom, Czaja, and Dutton (2008) found that adults who experienced child maltreatment are more vulnerable to experience re‐victimising events such as physical or sexual assault, but not more likely to experience other traumatic events such as disasters or accidents compared to non‐maltreated adults. Re‐victimisation thus describes the path from interpersonal trauma in childhood to interpersonal trauma in adulthood. The pathways from child maltreatment to re‐victimisation involve psychosocial and psychopathological components (Miron & Orcutt, 2014). However, it is unclear whether adult psychopathology is mainly associated with the experience of child maltreatment or with the experience of adult re‐victimisation, or both forms of interpersonal trauma (Cloitre et al., 2009).

In the present study, we aimed to investigate the path from child maltreatment to ICD‐11 CPTSD and examine the mediating role of different aspects of ER and adult re‐victimisation in this relationship. We hypothesised that child abuse and neglect would be associated with different aspects of ER and would both predict re‐victimisation. While child abuse is characterised by a more invasive disruption, neglect represents a deficit of adequate response by the environment. We therefore hypothesised that abuse would predict aspects of ER characterised by emotional flooding, while neglect would predict aspects of ER related to suppressing emotions. We further assumed that re‐victimisation would predict both, PTSD and DSO. We hypothesised that PTSD symptoms would be more directly predicted by child maltreatment and re‐victimisation, while DSO would be more mediated by ER.

## 2 METHODS

### 2.1 Participants and procedure

Participants in this study were individuals who were referred by general practitioners, psychiatrists or psychologists for psychological therapy to a National Health Service (NHS) trauma centre in Scotland. All 230 new patients over the 18‐month recruitment period were sent a letter and invited to complete a set of standardised measures. Twenty‐two did not respond and 13 provided unusable data due to large amounts of missing responses, and 2 had missing scores on the ICD‐TQ, which resulted in a final sample size of 193. The mean age of the sample was 40.7 years (*SD* = 12.4) and there were more women (65.1%) than men. Most of the sample were born in the United Kingdom (88.7%) and of these most were from Scotland (79%). The highest level of academic attainment was varied: school (38.5%), College (30.2%) and University (30.2%). Approximately a third of the sample was in employment (full‐time 20.2%, part‐time 13%), 38.9% were unemployed, 7.3% were retired and 5.7% were in voluntary work (15% reported ‘None of these’). Almost half of the sample were single (48.2%), 22.3% were married, 12.4% were divorced and 9.8% were co‐habiting. Most participants were either living with partner or with their family (41%), 34.7% were living alone (and 24.4% reported ‘Other’).

### 2.2 Materials

#### 2.2.1 Childhood trauma questionnaire

The childhood trauma questionnaire (CTQ; Bernstein & Fink, 1998) is a 28‐item self‐report questionnaire that assesses exposure to a range of different interpersonal childhood traumas. It yields five subscales, each with five items: emotional abuse, physical abuse, sexual abuse, emotional neglect and physical neglect. Items are responded to using a 5‐point scale ranging from ‘never true’ (1) to ‘very often true’ (5), which produces possible scores of 5–25 for each trauma subscale. In the present study, we combined the three abuse subscales to an abuse total scale and the two neglect subscales to a neglect total scale. The reliability of the abuse and neglect total scales was high in this sample; abuse (0.94), and neglect (0.92).

#### 2.2.2 Life‐events checklist for DSM‐5

The life‐events checklist (LEC) (Weathers et al., 2013) is a 17‐item self‐report measure designed to screen for potentially traumatic events in a respondent's lifetime, rated as either present or absent. In the present study, we only used those four items that assess re‐victimising interpersonal life events (two items assessing physical assault with or without a weapon and two items assessing sexual assaults or other unwanted or uncomfortable sexual experiences). This produced a single ‘total re‐victimisation’ variable with possible scores ranging from 0 to 4.

#### 2.2.3 International trauma questionnaire

The international trauma questionnaire (ITQ; version 1.2; Cloitre, Roberts, Bisson, & Brewin, 2013) is a 23‐item self‐report measure for proposed ICD‐11 PTSD and CPTSD diagnoses. Seven items are used to measure the three cluster of PTSD. CPTSD includes PTSD as well as three clusters reflecting DSO. Sixteen items represent the three clusters of affect dysregulation, negative self‐concept and disturbed relationships. Symptom endorsement for all items is scored on a scale ranging from ‘not at all’ (0) to ‘extremely’ (4). The scale can be used to generate a self‐report ICD‐11 PTSD or CPTSD diagnosis. The ICD‐11's taxonomic structure means that an individual can only be diagnosed with PTSD or CPTSD, not both. The psychometric properties are described elsewhere (Karatzias et al., 2016). The reliability of the PTSD and the DSO subscale was adequate in this sample at 0.77 and 0.94, respectively.

#### 2.2.4 Difficulties in emotional regulation scale

The difficulties in emotional regulation scale (DERS; Gratz & Roemer, 2004) is a standardised 36‐item measure of emotional dysregulation involving not just the modulation of emotional arousal, but also the awareness, understanding, and acceptance of emotions, and the ability to act in desired ways regardless of emotional state. It provides six subscales including ‘non‐acceptance of emotional responses’ (non‐accept), ‘difficulties in engaging in goal directed behaviour’ (goals), ‘impulse control difficulties’ (impulse), ‘lack of emotional awareness’ (aware), ‘limited access to emotional regulation strategies’ (strategies), and ‘lack of emotional clarity’ (clarity). Participants are asked to indicate how often the items apply to themselves, with responses ranging from ‘almost never’ (1) to ‘almost always’ (5). The total scale score was used in this study to reflect the overall degree of emotion dysregulation. The reliability of the subscales was high in this sample; non‐accept (0.90), goals (0.87), impulse (0.89), awareness (0.80), strategies (0.88), and clarity (0.73).

### 2.3 Statistical analysis

We used descriptive statistics to describe the research sample and correlational analysis to assess the bivariate relationship of all variables included in the path model. Since we used subscales of the questionnaires in the final path model and pre‐specified subscales might not hold in any given sample, we used confirmatory factor analysis (CFA) to investigate the construct validity of the scales in our sample. We used the weighted least squares means and variance adjusted (WLSMV) estimator for robust parameter estimation and conducted the CFA utilising the R‐package lavaan (Rosseel, 2012).

Path analysis was used to assess possible direct and indirect effects from childhood trauma on current post‐traumatic psychopathology. Different aspects of ER and adult re‐victimisation acted as possible mediators. The path model was specified using robust maximum likelihood estimation using the R‐package lavaan (Rosseel, 2012). Missing values were treated with the full information maximum likelihood method that is implemented in lavaan. Confidence intervals of the mediation were calculated by bootstrapping technique (number of bootstraps = 5,000).

For model fit criteria, we used the comparative fit index (CFI), the Tucker–Lewis Index (TLI) and the root mean square error of approximation (RMSEA), and applied the commonly used benchmarks TLI > 0.95, CFI > 0.95 and RMSEA < 0.05 as indicative of good model fit, and TLI > 0.90, CFI > 0.90 and RMSEA < 0.08 as indicative of acceptable model fit (Hu & Bentler, 1999; Kline, 2016).

## 3 RESULTS

### 3.1 Construct validity of subscales

The CTQ model was defined as a five first‐order factors and two second‐order factors model. All five types of abuse and neglect (physical abuse, sexual abuse, emotional abuse, physical neglect and emotional neglect) were used as first‐order factors with five items loading on each factor and the three types of abuse and the two types of neglect formed one higher order factor each. The resulting model had satisfactory model fit: TLI = 0.986, CFI = 0.988, RMSEA = 0.080, 90% CI = [0.071, 0.089]. The DERS factor model was defined as a six‐factor model. All items were specified to load on their respective scale, all scales were allowed to correlate within the model. The resulting model had satisfactory model fit: TLI = 0.912, CFI = 0.919, RMSEA = 0.074, 90% CI = [0.068, 0.081]. The factor structure of the ITQ in the present sample was analysed in previous work and the model with two second‐order factors representing PTSD and DSO showed good fit (Karatzias et al., 2016).

### 3.2 Trauma histories and zero‐order analyses

Scores from the CTQ indicated that there were high levels of childhood trauma, particularly emotional abuse and emotional neglect: mean (*SD*): emotional abuse, 14.20 (6.67); physical abuse, 10.76 (5.89); sexual abuse, 12.44 (8.07); emotional neglect, 13.48 (6.22) and physical neglect 9.53 (5.01). Endorsement rates for any item (score > 1) from the CTQ subscales indicated that any experience of childhood trauma was high: emotional abuse, 84.6%; physical abuse, 63.8%; sexual abuse, 53.3%; emotional neglect, 79.8% and physical neglect, 68.6%. The reported mean number of re‐victimising traumatic events using four items of the life events checklist was 2.36 (*SD* = 1.27). The endorsement rates to the events were 78.4% ‘physical assault’, 57.9% ‘sexual assault (rape, attempted rape, made to perform any type of sexual act through force or threat of harm)’, 50.7% ‘assault with a weapon’, and 48.2% ‘other unwanted or uncomfortable sexual experience’. The criteria for PTSD based on the ITQ were met in 90.1% of the sample. Taken into account the ICD‐11's taxonomic structure meaning that an individual can only be diagnosed with PTSD or CPTSD, not both, the self‐report prevalence of ICD‐11 PTSD and CPTSD were 37% and 53.1%, respectively.

Table 1 shows the descriptive statistics and the zero‐order correlations of all variables of interest. All correlation indices were positive and many substantial. We thus decided to include all variables in the path model.

**Table 1:** Bivariate correlations, means and standard deviations of variables included in path model

	M	*SD*	1	2	3	4	5	6	7	8	9	10	11
1. PTSD	20.2	5.4	‐	0.55	0.36	0.19	0.31	0.28	0.36	0.40	0.13	0.39	0.26
2. DSO	39.0	13.8		‐	0.51	0.43	0.45	0.44	0.59	0.62	0.22	0.72	0.48
3. Child abuse	37.0	17.5			‐	0.72	0.67	0.21	0.25	0.27	0.16	0.38	0.22
4. Child neglect	23.1	10.5				‐	0.49	0.05	0.27	0.21	0.22	0.28	0.27
5. Adult re‐victimisation	2.4	1.3					‐	0.14	0.29	0.27	0.10	0.29	0.20
6. ER non‐accept	20.2	7.2						‐	0.36	0.48	0.22	0.56	0.39
7. ER goals	19.7	4.8							‐	0.56	0.12	0.65	0.40
8. ER impulse	17.0	6.4								‐	0.25	0.63	0.51
9. ER aware	19.2	5.8									‐	0.20	0.46
10. ER strategies	26.6	7.7										‐	0.40
11. ER clarity	14.8	4.4											‐

### 3.3 Path analysis

**Figure 1:**
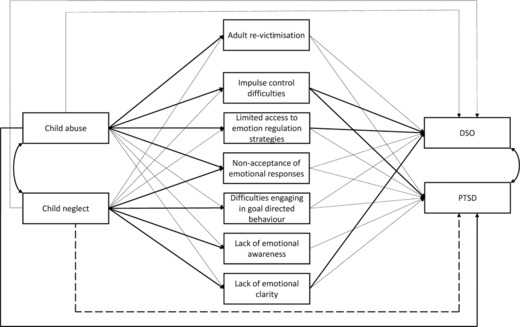
Path model from child maltreatment to post‐traumatic psychopathology via emotion regulation difficulties and re‐victimisation *Notes*. Positive significant associations are printed as solid bold line, negative significant associations are printed as dashed bold line.

The path model is displayed in Figure 1 and the results are summarized in Table 2. The model demonstrated good fit as measured with all fit indices: *χ*²(*df* = 6) = 7.98, *P* = 0.240, CFI = 0.998, TLI = 0.979, RMSEA = 0.041, 95% CI = [<0.001, 0.107]. The model explained 64.2% of the variability of the DSO symptoms (*R*² = 0.642) and 26.5% of the variability of the PTSD symptoms (*R*² = 0.265). The inter‐correlation of the two predictor variables (child abuse and child neglect) was *r* = 0.72, the inter‐correlation between the DERS subscales varied from *r* = 0.08 to 0.64, and the inter‐correlation of the two outcome variables (PTSD and DSO) was *r* = 0.32. PTSD symptoms were significantly predicted by child abuse and by impulse. Interestingly, child neglect negatively predicted PTSD symptoms, although the bivariate correlation between child neglect and PTSD was positive (*r* = 0.19, see Table 1). DSO symptoms were predicted by impulse, strategies and clarity. Adult re‐victimisation neither predicted PTSD nor DSO, but was predicted by child abuse. Child abuse and child neglect differentially predicted DERS subscales: impulse and strategies were predicted by child abuse; goals, aware and clarity were predicted by child neglect. Non‐accept was predicted by both, child abuse and neglect. Four single mediation effects were statistically significant (Table 3). Taken all DERS scales together, there was a significant mediation effect from child abuse to DSO via ER, no other mediation effect from child abuse or neglect to PTSD or DSO was significant (Table 3). The total effects from child abuse to PTSD (estimator = 0.26, *SE* = 0.06, 95% CI = [0.15, 0.39]) and DSO (estimator = 0.21, *SE* = 0.09, 95% CI = [0.03, 0.39]) via ER were significant. The total effect from child neglect to PTSD via ER was significant (estimator = 0.24, *SE* = 0.08, 95% CI = [0.09;0.42]), while the total effect from child neglect to DSO via ER was not. Overall results indicate that the path from child maltreatment to PTSD is a direct one, while the path to DSO is mediated by ER and therefore an indirect one.

**Table 2:** Path coefficients of mediation model predicting the dimensions of CPTSD (PTSD and DSO)

	*β*	*b*	*SE*	*z*	*P*
PTSD					
Trauma					
Child abuse	0.32	0.21	0.06	3.47	0.001
Child neglect	−0.19	−0.14	0.06	−2.27	0.023
Adult re‐victimisation	0.09	0.05	0.05	1.06	0.290
Emotion regulation					
Non‐accept	0.02	0.01	0.05	0.21	0.834
Goals	0.15	0.12	0.08	1.52	0.129
Impulse	0.17	0.13	0.06	2.01	0.045
Aware	0.02	0.02	0.06	0.31	0.759
Strategies	0.07	0.05	0.08	0.64	0.521
Clarity	0.03	0.03	0.07	0.39	0.700
DSO					
Trauma					
Child abuse	0.12	0.09	0.06	1.50	0.133
Child neglect	0.08	0.07	0.06	1.17	0.241
Adult re‐victimisation	0.11	0.08	0.04	1.83	0.068
Emotion regulation					
Non‐accept	0.03	0.03	0.04	0.58	0.559
Goals	0.09	0.08	0.06	1.31	0.189
Impulse	0.18	0.14	0.05	3.10	0.002
Aware	−0.01	−0.01	0.05	−0.27	0.789
Strategies	0.38	0.34	0.07	5.24	<0.001
Clarity	0.12	0.12	0.06	2.05	0.040

*β*: standardised path coefficient; *b*: un‐standardised coefficient; *SE*: standard error, *z*: *z*‐statistic, *P* = *P*‐value.

**Table 3:** Significant indirect effects

Path	Estimator	*SE*	95% CI
Abuse → strategies → DSO	0.10	0.04	[0.04, 0.19]
Abuse → impulse → PTSD	0.03	0.02	[< 0.01, 0.08]
Abuse → impulse → DSO	0.03	0.02	[< 0.01, 0.08]
Neglect → clarity → DSO	0.02	0.02	[< 0.01, 0.07]
Abuse → ER → DSO	0.15	0.05	[0.05, 0.25]

*Note*. 95% CI based on 5,000 bootstraps.

## 4 DISCUSSION

In the current study, we aimed to investigate the path from child maltreatment CPTSD and to examine the mediating role of ER and adult interpersonal re‐victimisation in this relationship. We found a differential relation between the types of child maltreatment, aspects of ER and the two dimensions of CPTSD (PTSD and DSO). Overall, the data supported the basic idea that ER mediates the association of child maltreatment with CPTSD symptom distress (Choi et al., 2014; Stevens et al., 2013).

### 4.1 Child maltreatment and re‐victimisation

Contrary to our expectation but similarly to Cloitre et al. (2009), adult interpersonal re‐victimisation did not predict PTSD or DSO symptoms in the path model, although the bivariate correlations were substantial in size. The experience of child maltreatment and the levels of different aspects of ER accounted for the variability introduced by adult re‐victimisation, indicating that child maltreatment has a more powerful negative association with adult mental health than traumatisation in adulthood for those who experience trauma during both times in their lives. Maltreated children very often experience more types of abuse and neglect. The effect of this poly‐victimisation accounts to a large extant for the deleterious effects of child maltreatment (Cecil, Viding, Fearon, Glaser, & McCrory, 2017). As we considered different forms of interpersonal trauma including child abuse and neglect as well as adult re‐victimisation in one model predicting adult psychopathology, the cumulative effect of all types of interpersonal traumatic experiences exceeded the effects of adult re‐victimisation. Interestingly and contrary to our hypothesis, neglect negatively predicted PTSD symptoms in the path model. Similar to re‐victimisation, child abuse and neglect both were positively correlated with each other and with the dimensions of CPTSD (PTSD and DSO) in the bivariate analysis. It seems that the positive shared variance between neglect and PTSD can be fully explained by the other variables in the model. This was also found in previous research investigating the differential associations of types of abuse and neglect and PTSD symptomatology (Lueger‐Schuster et al., 2018) and can be explained by the type of the traumatic events: Neglect is characterised by the absence of adequate environmental response to the developmental needs of a child, which is not an event as such that could be re‐experienced or avoided. However, in the present study, more neglect predicted fewer symptoms of PTSD in the path model. A possible explanation might be that neglect is associated with emotional numbing, which is not part of ICD‐11 PTSD, and that some of the ICD‐11 PTSD symptoms such as hyper‐arousal are not experienced as a problem by adults who tend to feel numb. Future research should address the impact of specific types of interpersonal re‐victimisation, such as domestic violence, and investigate if there are characteristic paths from certain types of child maltreatment to certain types of re‐victimisation. Furthermore, the role of neglect for adult psychopathology needs to be addressed in future research.

### 4.2 Emotion regulation as mediator

As hypothesised, aspects of ER were differentially predicted by child abuse and neglect. Difficulties remaining in control of one's behaviour when experiencing negative emotions (impulse) and the belief that there is little that can be done to regulate emotions effectively, once an individual is upset (strategies) were predicted only by child abuse. Both scales reflect the individual's response to the presence of intense emotions. Being overwhelmed by emotions and not having effective response mechanisms might be rooted in abusive childhood experiences that were accompanied by intense negative emotions that could not be regulated. In contrast, child neglect predicted aspects of ER that can be described as muted; inattention to, and lack of awareness of, emotional responses (aware), a lack of clarity about the emotions they are experiencing (clarity) and difficulties concentrating and accomplishing tasks when experiencing negative emotions (goals). This result corroborates previous findings (Berzenski, 2018) and also is consistent with research showing a stronger association of child abuse with externalising and neglect with internalising problems (Petrenko et al., 2012). Neglect is defined as deficits in meeting the basic needs of a child and characterised by omission rather than commission (Erickson & Egeland, 2002), which seems to be mirrored in more passive and restrained aspects of ER (Berzenski, 2018; Hildyard & Wolfe, 2002).

### 4.3 Clinical and research implications

Our results emphasise the pivotal role of ER in the relationship of traumatic experiences and psychopathological distress. Notably, ER is stronger associated with DSO than with PTSD, suggesting that ER could be more important for the development and maintenance of DSO than of PTSD symptoms. Even though established treatment protocols for PTSD seem to work for CPTSD as well (Jongh et al., 2016), strengthening adaptive ER strategies might further facilitate recovery from child maltreatment related psychopathology (Cloitre et al., 2010). More general, trauma‐specific interventions not designed to target ER also result in increased ER abilities regardless of a history of child maltreatment (Jerud, Zoellner, Pruitt, & Feeny, 2014), suggesting that ER is a closely related construct to PTSD and CPTSD. Future research should address the potentially beneficial effect of treatment protocols explicitly aiming at reducing dysfunctional ER strategies and compare them to standard treatment protocols. Symptoms of the DSO clusters might particularly be addressed with interventions aiming to strengthen the belief that there is something that can be done to regulate emotions effectively when people are overwhelmed with negative emotions and to support clarity about the emotions an individual is experiencing. Further research should also investigate the relation of ER and re‐victimisation. It is possible that specific dysfunctional aspects of ER will place individuals at higher risk of becoming victim to interpersonal violence in adulthood than others. Possibly, a lack of emotional awareness and clarity might lead to unfavourable interpersonal relationships that in turn are associated with higher risk of intimate partner violence.

### 4.4 Limitations

This study has some limitations that need to be considered. First, the data are cross‐sectional and thus causal conclusions cannot be drawn. It may also be the case that ER and post‐traumatic symptoms mutually predict each other, however, in the current study, we emphasised ER as possible mediator between child maltreatment and symptom distress. ER and DSO also share conceptual and statistical variance that accounts for parts of for their association. Longitudinal studies should investigate the relationship of ER and DSO to disentangle their causal connection. Second, the ITQ is a new scale and requires further validation with various samples exposed to a variety of traumatic stressors. Third, we assessed child maltreatment retrospectively and thus had to rely on participant's recollections from childhood, which might be biased. Fourth, we did not assess witnessing of domestic violence in the present study that often is referred to as distinct type of trauma in childhood, besides abuse and neglect. Including different types of interpersonal trauma, such as witnessing domestic violence, might result in different association patterns. Finally, the model tested in this study needs further replication in different and possibly larger samples before its results can be generalised to other groups.

### 4.5 Conclusion and outlook

Notwithstanding its limitations, our results suggest that the path from child maltreatment to ICD‐11 PTSD is a direct one, while the path to DSO is mediated by ER and therefore an indirect one. However, the pathogenic mechanisms underlying the differential associations of interpersonal trauma such as child abuse, neglect, and adult re‐victimisation are not yet fully understood. Future research is required to look especially into the role of neglect and re‐victimisation in the development of adult post‐traumatic psychopathology. Finally, research should investigate the potential effects of strengthening functional ER strategies as supplementary treatment option in adults with histories of child maltreatment (Cloitre, Koenen, Cohen, & Han, 2002) and the potential effect that this might have on their DSO symptoms.
